# *Limosilactobacillus reuteri*‐ *Fusobacterium nucleatum* Interactions Modulate Biofilm Composition and Immunogenicity

**DOI:** 10.1111/jre.70021

**Published:** 2025-07-25

**Authors:** Luming Yang, Gopu Sriram, Ren Jie Jacob Chew, Kai Soo Tan

**Affiliations:** ^1^ Faculty of Dentistry National University of Singapore Singapore; ^2^ Oral Care Health Innovations and Designs Singapore National University of Singapore Singapore

**Keywords:** biofilm, immunogenicity, *L. reuteri*, microbiology, *P. gingivalis*, periodontitis, probiotics

## Abstract

**Aim:**

The interactions between *Limosilactobacillus reuteri* and oral bacteria are poorly understood. This study seeks to characterize how two strains of *L. reuteri* coaggregate with *Fusobacterium nucleatum* , determining the impact on the biofilm composition and immunogenicity.

**Methods:**

A series of in vitro experiments was conducted using *L. reuteri* DSM 17938 and ATCC PTA 5289, *Fusobacterium nucleatum* ATCC 25586, and *Porphyromonas gingivalis* W50. The coaggregation between individual strains of *L. reuteri* , *F. nucleatum* , and *P. gingivalis* was evaluated using the tube coaggregation assay and confocal microscopy. Biofilm compositions were determined by confocal microscopy and culture. The effect of coaggregation on the immunogenicity of *L. reuteri‐F. nucleatum* aggregates were evaluated using periodontal ligament fibroblasts, oral epithelial cells, and monocytes.

**Results:**

Both *L. reuteri* DSM and PTA strains demonstrated coaggregation with *F. nucleatum* . This interaction reduced the amount of *F. nucleatum* in biofilm by 1000‐fold. Additionally, the coaggregation between *L. reuteri* and *F. nucleatum* lowered its immunogenicity. Furthermore, the coaggregation of *L. reuteri* with *F. nucleatum* led to a 50% reduction in the amount of *P. gingivalis* present in the biofilm.

**Conclusion:**

This study demonstrates novel mechanisms through which *L. reuteri* can exert its effects as a probiotic. The coaggregation with *L. reuteri* modulates the immunogenicity of *F. nucleatum* and impairs its ability to serve as the bridging species, altering the biofilm composition, thus limiting the extent of dysbiosis.

## Graphical abstract

The coaggregation between *L. reuteri* and *F. nucleatum* modulates its immunogenicity and affects the latter's ability to coaggregate with late colonizers, altering the biofilm composition.

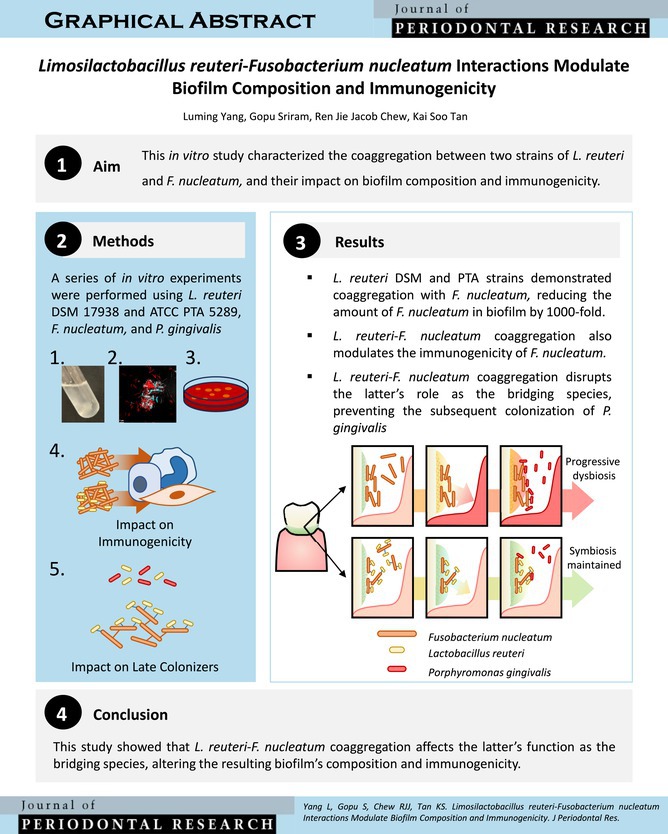

**Summary**

- Background
  ○ Although *Limosilactobacillus reuteri* is adjunctively used with nonsurgical periodontal treatment, its effects are variable.
  ○ Studies evaluating its underlying mechanisms have focused on its secreted antimicrobial compounds; thus, its interactions with oral bacteria are poorly understood.
- Added value of this study
  ○ This study characterized the coaggregation between two strains of *L. reuteri* and *Fusobacterium nucleatum* , limiting the amount of *F. nucleatum* present in biofilms.
  ○
*L. reuteri* ‐ *F. nucleatum* coaggregation also modulates the immunogenicity of *F. nucleatum* , translating to reduced host inflammatory responses.
  ○ These effects were independent of secreted antimicrobial compounds such as reuterin, which were well‐characterized mechanisms underlying the effects of *L. reuteri* .
  ○
*L. reuteri* ‐ *F. nucleatum* coaggregation also disrupts the latter's role as the bridging species, preventing the subsequent colonization of *Porphyromonas gingivalis* .
- Clinical implications
  ○ The coaggregation between *L. reuteri* and other oral bacteria may be another mechanism through which *L. reuteri* can exert its effects as a probiotic.
  ○ Coaggregation between *L. reuteri* and *F. nucleatum* may limit the immunogenicity and dysbiotic changes in the subgingival biofilm.
  ○ Since coaggregation is a fundamental interaction between bacteria, further characterization of the coaggregation between *L. reuteri* and other oral bacteria may help improve its clinical use as a periodontal probiotic.

## 1 Introduction

Probiotics have been used adjunctively with nonsurgical periodontal therapy for more than a decade, yet the clinical benefits vary significantly depending on the specific species or strain used, ranging from no clinical impact to notable improvements of periodontal parameters [1]. Significant improvements have primarily been reported in studies evaluating *Limosilactobacillus reuteri*, formerly known as *Lactobacillus reuteri* [2, 3]. However, these clinical benefits were often short‐lived, typically lasting around 3 months, and the extent of improvement varies across different studies [4, 5]. Understanding the underlying mechanisms and effects of *L. reuteri* on oral bacteria and immune response may offer new insights into optimizing the use of probiotics in periodontal therapy, addressing the variable clinical efficacy.

*L. reuteri* is often administered in the form of lozenges containing *L. reuteri*, DSM 17938, and ATCC PTA 5289. Clinically, adjunctive *L. reuteri* reduces the re‐colonization of key periodontal pathogens such as *Porphyromonas gingivalis* , minimizing inflammation and tissue destruction [2, 3, 6]. These benefits are primarily attributed to various bioactive compounds secreted by *L. reuteri* , particularly reuterin [7, 8, 9].

Heat‐inactivated *L. reuteri* and its culture supernatant have both been shown to affect the viability of periodontal pathogens [7, 10, 11], suggesting an additional mechanism beyond the secretome of *L. reuteri* . An alternative perspective involves exploring the interactions, such as the coaggregation between *L. reuteri* and other oral microbes, which are fundamental to biofilm development. However, while studies have demonstrated how the different strains of *L. reuteri* exhibited strain‐specific coaggregation with gut pathogens [12], there is a lack of research on how *L. reuteri* DSM 17938 and ATCC PTA 5289 strains interact with oral microbes. In the context of periodontitis, *Fusobacterium nucleatum* is a significant microbe that connects early and later colonizers, facilitating the colonization of periodontal pathogens, including *P. gingivalis* . Therefore, the objective of this study is to characterize the interactions between the *L. reuteri* DSM and PTA with *F. nucleatum* , assessing their impact on the biofilm composition and immunogenicity.

## 2 Methods

### 2.1 Biofilm Culture

*L. reuteri* DSM 17938 and ATCC PTA 5289 (BioGaia, Stockholm, Sweden), *Fusobacterium nucleatum* ATCC 25586, and *Porphyromonas gingivalis* W50 (American Type Culture Collection, Virginia, USA) were used in this study. Detailed bacterial culture conditions are described in the Supporting Information. Biofilms were cultured in 24‐well plates (Thermo Fisher Scientific, Massachusetts, USA) with *F. nucleatum* (1 × 10^7^ CFU) and *L. reuteri* (1 × 10^7^ and 5 × 10^7^ CFUs) using BHI broth with 0.5% yeast extract, 0.05% hemin, and 0.0001% menadione and incubated anaerobically at 37°C for 24 h. The viability of *F. nucleatum* was evaluated using selective agar plates, which consisted of BHI agar supplemented with 0.5% yeast extract (Neogen), 0.05% hemin, 0.0001%, 5 mg/L Vancomycin (Merck), 100 mg/L Neomycin (Merck), and 3 mg/L Josamycin (Merck).

### 2.2 Coaggregation Assay

Coaggregation assay was performed as described previously with modifications [13, 14]. For the visual coaggregation assay, overnight bacterial cultures were pelleted by centrifugation and washed twice in 1× PBS. Then, the required bacterial partners were adjusted to an optical density of 600 nm (OD 600 nm) of 1, mixed in a clear glass test tube, and incubated at room temperature for 1 h. Quantitative coaggregation assay was set up in the same manner as the visual coaggregation assay except that the supernatant containing unaggregated cells was transferred to a flat‐bottom 96‐well plate (Thermo Fisher Scientific), and the optical density at 600 nm was measured. The percentage of the coaggregation was determined as follows:

$$\%\text{coaggregation}=\left({\mathrm{OD}}_{0}-{\mathrm{OD}}_{1}\right)/{\mathrm{OD}}_{0}\times 100$$

OD_0_ is the absorbance of the initial mixture at 0 min, and OD_1_ is the absorbance of the supernatant at 1 h time‐point.

### 2.3 Confocal Laser Scanning Microscopy (CLSM)

Overnight bacterial cultures were pelleted by centrifugation and washed once with sterile 1× PBS. The absorbance was adjusted to optical density (OD) 1 at 600 nm and resuspended in fresh media containing Hydroxycoumarin‐amino‐D‐alanine (HADA‐blue) and Red TAMRA‐amino‐D‐alanine (Rada‐red) fluorescent probes (Tocris, Bio‐Techne, Minneapolis, USA) at a final concentration of 20 mM. The cultures were incubated in the dark for 2 h under appropriate growth conditions, centrifuged to remove supernatant, washed twice with 1× PBS, and mixed with their coaggregation‐paired bacterial species. Subsequently, an aliquot of the bacterial aggregate was placed on a glass slide and examined using CSLM (Olympus FV 3000, Evident Corporation, Tokyo, Japan).

For evaluation of biofilm by CLSM, *F. nucleatum* biofilms were inoculated at 1 × 10^8^ CFU and cultured alone or together with *L. reuteri* DSM or PTA strains at a ratio of 1:1 and 1:5. *F. nucleatum* was labeled using the fluorescence dye HADA, while DSM or PTA were labeled with RADA at a concentration of 20 mM. *F. nucleatum‐P. gingivalis* biofilms were inoculated at 1 × 10^8^ CFU for each and cultured in the presence or absence of *L. reuteri* DSM or PTA strains at a ratio of 1:1. *F. nucleatum* and *P. gingivalis* were labeled with HADA, while DSM or PTA were labeled with RADA at a concentration of 20 mM. The biofilms were incubated in 4‐well chambered coverslips (Ibidi, Gräfelfing, Germany) anaerobically in the dark for 6 h. After incubation, the 3D images of biofilms were captured with CLSM and analyzed using Imaris 10.0 software (Oxford Instruments, Oxford, United Kingdom).

### 2.4 Spot Assay

Spot assay was performed as described previously [15]. *L. reuteri* was spotted on BHI agar supplemented with 0.5% yeast extract (Neogen), 0.05% hemin (Merck), 0.0001% menadione (Merck), and incubated for 24 h either aerobically or anaerobically. Each spot consisted of 7 μL of 1 × 10^9^ CFU/mL of the overnight culture. *F. nucleatum* was then spotted next to the *L. reuteri* and incubated for another 24 h anaerobically.

### 2.5 Small Molecule Screening

The specificity of the adhesin‐receptor interactions between *F. nucleatum* and *L. reuteri* was characterized through the screening of small molecules, which consisted of either sugars or amino acids, to identify molecules that mimic the structure of either the adhesin or the receptor, thus blocking the interaction and preventing bacterial coaggregation (Table S1). A quantitative coaggregation assay was performed as described above in the presence or absence of the small molecule of interest. The tested small molecule was dissolved in coaggregation buffer and adjusted to a pH of 7.4 before mixing the tested paired bacterial species for coaggregation.

### 2.6 Cell Culture and Treatment

Primary human periodontal ligament fibroblasts, PDLF (Science, California, USA), a human oral epithelial cell line, TR146 (European Collection of Authenticated Cell Cultures, Salisbury, United Kingdom), and 3 cell lines stably expressing nuclear factor‐κB‐ secreted embryonic alkaline phosphatase (NF‐κB‐SEAP) reporter were used in this study. These reporter cell lines included a human monocyte cell line (THP‐1 BLUE) and 2 human embryonic kidney (HEK) cell lines expressing either toll‐like receptor (TLR) 2 or TLR4 (Invivogen, California, USA). Detailed culture conditions are in the Supporting Information.

PDLF, TR146, HEK TLR2, and HEK TLR4 cells were seeded in 6‐well plates (Thermo Fisher Scientific) and allowed to adhere overnight. For infection with unconjugated bacteria, overnight cultures of *L. reuteri* and *F. nucleatum* were washed twice with 1× PBS and suspended in fresh cell culture media. The cells were infected with bacteria at a multiplicity of infection (MOI) of 50:1. The plates were incubated at 37°C with 5% CO_2_ for 6 h. For infection with bacterial aggregates, the respective bacterial species were aggregated in 0.2 mL tubes containing 50 μL of fresh cell culture media for 2 h. The formed aggregates were then transferred to the pre‐seeded cell culture plates and resuspended with additional fresh media using a wide‐bore tip, ensuring even distribution and effective interaction with the seeded cells. The plates were incubated under aerobic conditions for 6 h to facilitate bacterial infection.

### 2.7 Enzyme‐Linked Immunosorbent Assay (ELISA) and Reporter Assay

The amount of interleukin‐8 (IL‐8) protein in the culture supernatant was quantified using ELISA (Biolegend, California, USA). ELISA was carried out according to the protocol provided by the manufacturer. The net IL‐8 production was calculated by subtracting the cytokine levels in the uninfected control group. SEAP activity was determined using the Phospha‐Light assay kit (Thermo Fisher Scientific). The luminescence signal was measured using a GloMax Navigator plate reader (Promega, Wisconsin, USA). The results were expressed as fold change relative to the uninfected controls.

### 2.8 Statistical Analysis

All experiments were carried out in biological triplicate and repeated 3 times. Results were expressed as mean and standard deviation. The Shapiro–Wilk test for normality was first performed to confirm that the data were normally distributed. One‐way ANOVA was used for multiple comparisons with Tukey's or Dunnett's post hoc analysis. Statistical analyses were performed using GraphPad Prism version 10 (Massachusetts, USA), with statistical significance at *p* < 0.05.

## 3 Results

### 3.1 Coaggregation Between *L. reuteri* and *F. nucleatum* Influenced Biofilm Composition

**FIGURE 1:**
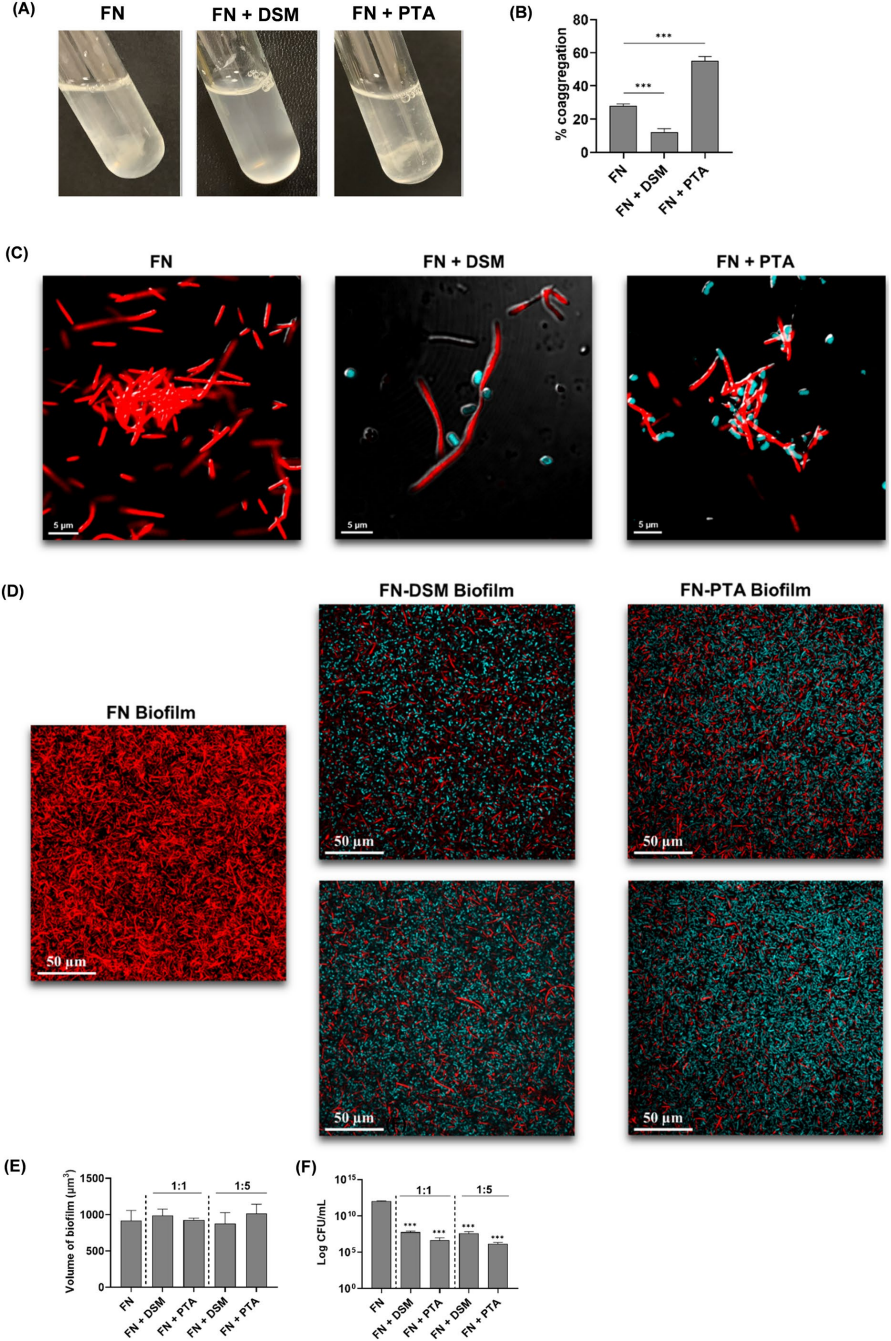
*L. reuteri* coaggregated with *F. nucleatum* . (A) Tube coaggregation assay of *F. nucleatum* (FN) alone, FN with *L. reuteri* DSM, and FN with *L. reuteri* PTA. (B) Quantitative analysis of the amount of coaggregates formed. CLSM analyses of (C) bacterial aggregates and (D) mono‐ or dual‐species biofilms. FN was labeled red using RADA while *L. reuteri* DSM or PTA strains were labeled blue using HADA. Quantification of the (E) biovolumes of various biofilms. (F) The amount of FN in biofilms was determined by plating on FN‐selective agar plates. The data shown are the mean results obtained from 3 independent experiments. ***p < 0.001 compared to FN alone.

To macroscopically assess whether *L. reuteri* can coaggregate with *F. nucleatum*, a tube coaggregation assay was performed. *F. nucleatum* exhibited self‐aggregation, forming visible clumps (Figure 1A). While bigger clumps were formed after introducing *L. reuteri* PTA, the mixture remained turbid without clump formation with *L. reuteri* DSM. This strain‐specific effect was consistent with quantitative analysis using optical density (Figure 1B). When microscopically examined, *L. reuteri* DSM coaggregated with *F. nucleatum* , preventing the self‐aggregation of *F. nucleatum* , resulting in the macroscopic observations (Figure 1C). In contrast, when *L. reuteri* PTA coaggregated with *F. nucleatum*, these aggregates coalesced with the clumps formed by *F. nucleatum* , resulting in the larger clumps observed. While both strains of *L. reuteri* can coaggregate with *F. nucleatum* , they result in different macroscopic presentations.

Considering the importance of coaggregation in oral biofilms [16], the impact of coaggregation with *L. reuteri* on the biofilm formation was evaluated. Both DSM and PTA strains integrated into the biofilms with no significant difference in biofilm volumes between the dual‐ and mono‐species biofilms (Figure 1D,E). When quantifying the amount of *F. nucleatum* in these dual‐species biofilms, we observed significant reductions in *F. nucleatum* levels in dual‐species biofilms compared to *F. nucleatum* mono‐species biofilm, regardless of the amount of *L. reuteri* present (Figure 1F).

### 3.2 Attenuating *L. reuteri* and *F. nucleatum* Coaggregation Reverses the Impact on Biofilm Composition

**FIGURE 2:**
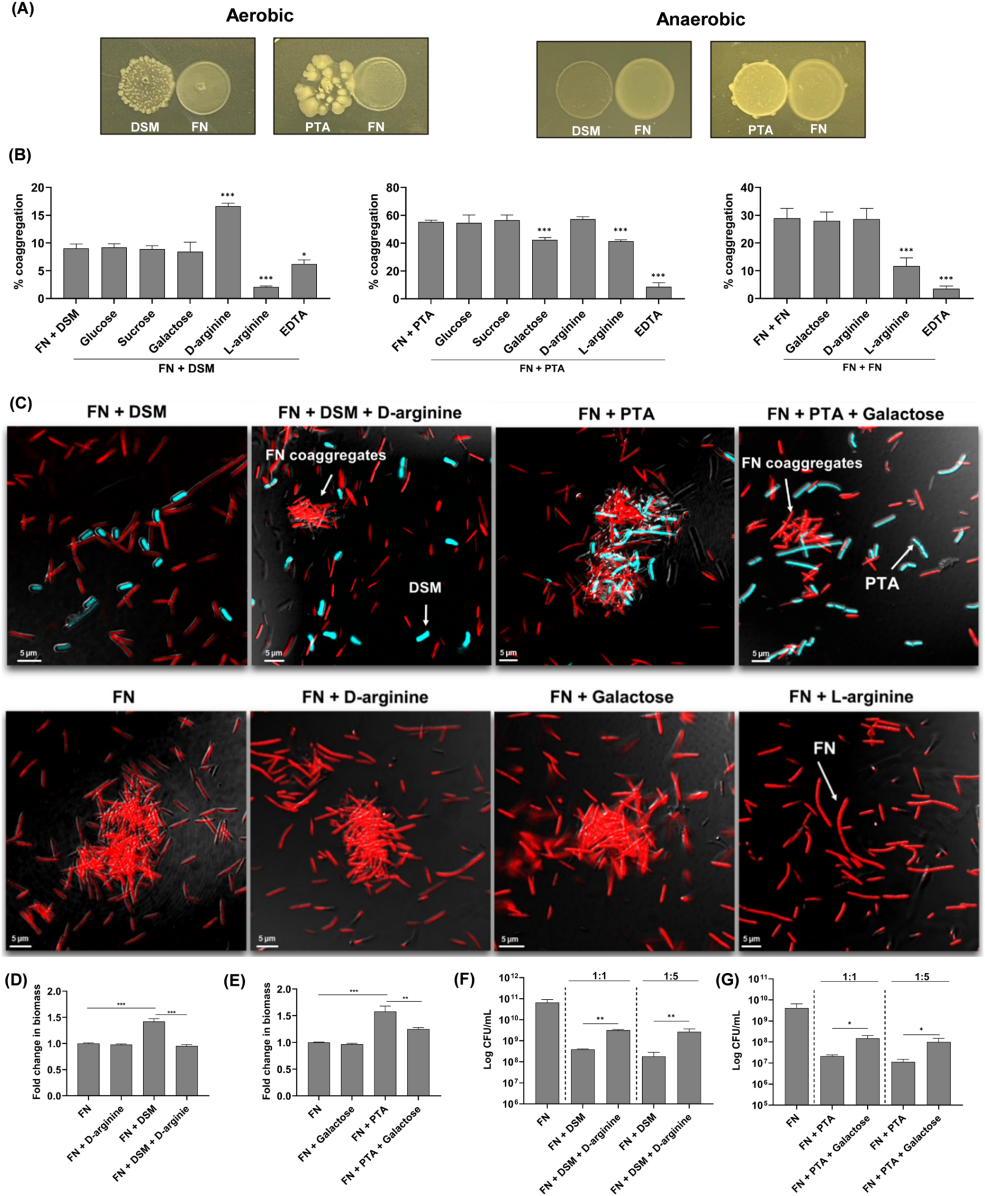
Impact of coaggregation on biofilm composition. (A) *L. reuteri* DSM or PTA strains were spotted on an agar plate and incubated for 24 h either aerobically or anaerobically. Thereafter, *F. nucleatum* (FN) was spotted next to the *L. reuteri* colony, and the plates were incubated anaerobically for 24 h. (B) Quantitative coaggregation assay or (C) CLSM analysis between FN and DSM; FN and PTA or FN alone carried out in the presence or absence of the indicated small molecule. FN was labeled red using RADA while *L. reuteri* DSM or PTA strains were labeled blue using HADA. (D, E) The biofilms were cultured in the presence or absence of D‐arginine or galactose for 24 h anaerobically, and biofilm biomass was quantified using the crystal violet assay. (F, G) The amount of FN in biofilms was determined by plating on FN‐selective agar plates. The data shown are the mean results obtained from 3 independent experiments. *p < 0.05 **p < 0.01; ***p < 0.001.

Spot assay was conducted to determine if the negative effect of *L. reuteri* on the viability of *F. nucleatum* could be attributed to secreted metabolites. Neither DSM nor PTA strains affected *F. nucleatum* viability when the probiotics were cultured under aerobic or anaerobic conditions (Figures 2A and S1A). While the antimicrobial effects of *L. reuteri* are most commonly attributed to acid or reuterin production [7, 8, 9], neither reuterin nor changes in pH were detected under current experimental conditions, suggesting that the reduced viability of *F. nucleatum* may be attributed to alternative mechanisms (Figure S1B,C).

To determine if this loss of viability was attributed to coaggregation, and independent of the secretome of *L. reuteri* , we attempted to replicate these interactions using heat‐killed *L. reuteri* . However, we observed a differential heat sensitivity of *L. reuteri‐F. nucleatum* coaggregation, where the adhesion‐receptors mediating interactions between *L. reuteri* DSM‐ *F. nucleatum* were heat sensitive, while *L. reuteri* PTA‐ *F. nucleatum* was heat‐insensitive (Figure S2). As an alternative, we sought to characterize the specificity of adhesin‐receptor interactions between *L. reuteri* strains and *F. nucleatum* . We found that the presence of D‐arginine, galactose, EDTA, or L‐arginine blocked *L. reuteri* ‐ *F. nucleatum* coaggregation (Figure 2B). However, it was essential to distinguish between small molecules that affect *F. nucleatum* self‐aggregation compared to those that specifically block *L. reuteri* ‐ *F. nucleatum* . We found that L‐arginine and EDTA reduced *F. nucleatum* self‐aggregates, suggesting they blocked *F. nucleatum* interactions. In contrast, D‐arginine specifically inhibited *F. nucleatum‐*DSM coaggregation. The amount of bacterial aggregates decreased when *F. nucleatum* and *L. reuteri* PTA coaggregated in the presence of either galactose, L‐arginine, or EDTA. Since L‐arginine and EDTA affected *F. nucleatum* self‐aggregation, galactose appeared to be a specific inhibitor for *F. nucleatum* ‐ *L. reuteri* PTA interactions. CLSM analysis confirmed that D‐arginine effectively blocked *L. reuteri* DSM and *F. nucleatum* coaggregation, and galactose inhibited the interaction between *L. reuteri* PTA and *F. nucleatum* (Figure 2C).

When D‐arginine or galactose was introduced into co‐cultures of DSM‐ *F. nucleatum* or PTA‐ *F. nucleatum* , respectively, their biomass reduced to levels similar to *F. nucleatum* monospecific biofilms (Figure 2D,E). This inhibition also decreased the amount of *F. nucleatum* in these dual‐species biofilms. Notably, the presence of D‐arginine or galactose led to an increase in viable *F. nucleatum* compared to co‐culturing in its absence (Figure 2F,G). Collectively, these findings highlight the important role of coaggregation in reducing *F. nucleatum* levels by *L. reuteri* in dual‐species biofilms.

### 3.3 Effects of Coaggregation on Innate Immune Response

**FIGURE 3:**
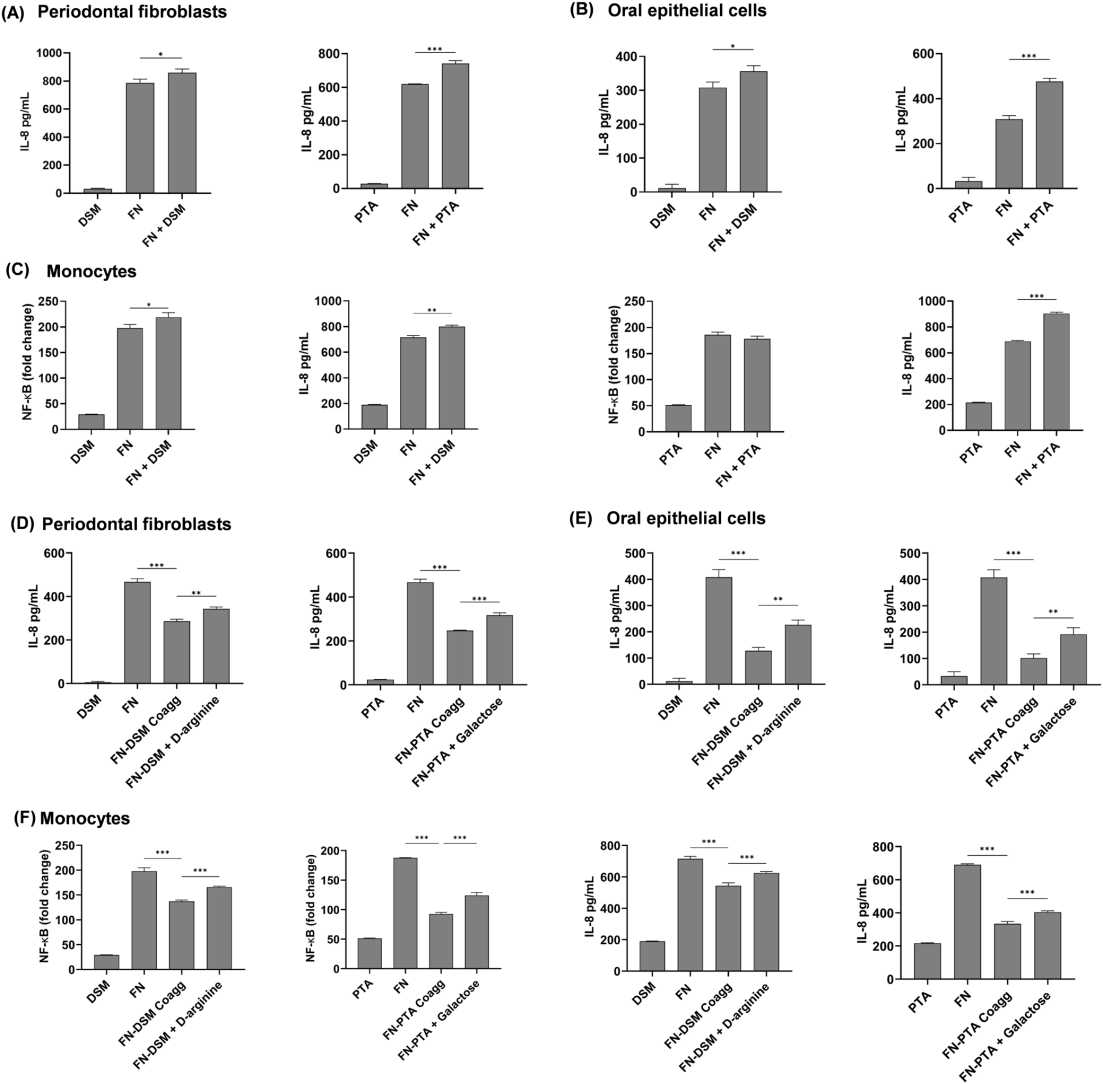
Effects of bacterial coaggregates on immune response. (A) Periodontal ligament fibroblasts, (B) oral epithelial cells, and (C) monocytes were infected with *L. reuteri* alone, *F. nucleatum* (FN), or unconjugated *L. reuteri* and FN. The amount of immune response was determined by measuring NF‐κB activation in monocytes and the secretion of IL‐8 by periodontal ligament fibroblasts, oral epithelial cells, and monocytes. FN + DSM or FN + PTA were coaggregated in the presence or absence of either D‐arginine or galactose before infecting (D) periodontal ligament fibroblasts, (E) oral epithelial cells, and (F) monocytes. The amount of immune response was determined by measuring NF‐κB activation in monocytes and the secretion of IL‐8 by periodontal ligament fibroblasts, oral epithelial cells, and monocytes. The data shown are the mean results obtained from 3 independent experiments. *p < 0.05; **p < 0.01; ***p < 0.001.

**FIGURE 4:**
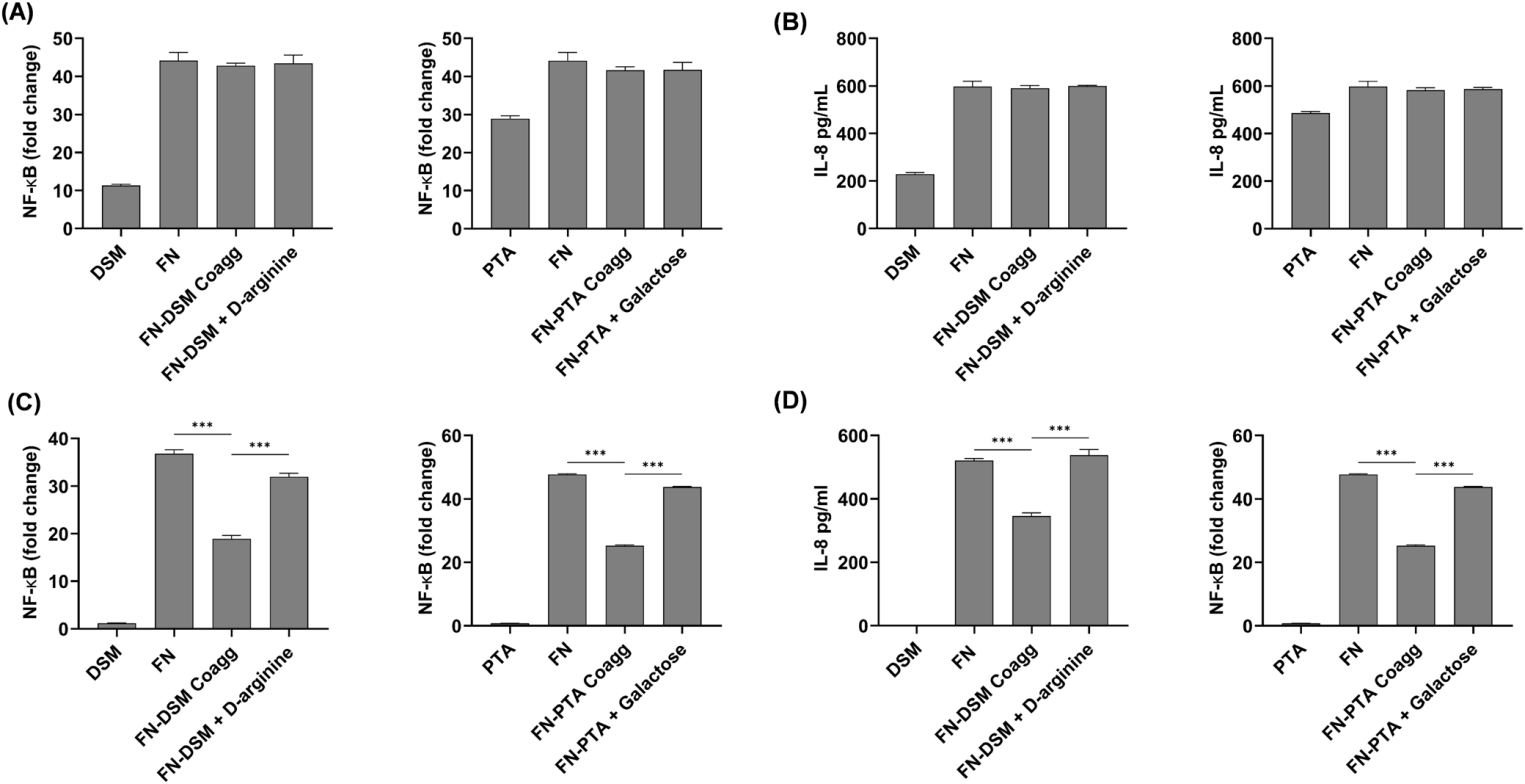
Bacterial coaggregates reduced toll‐like receptor (TLR) 4 activation. *F. nucleatum* (FN), FN, and DSM or FN and PTA were coaggregated in the presence or absence of either D‐arginine or galactose before infecting human embryonic kidney cells (HEK) expressing (A, B) TLR2 or (C, D) TLR4. TLR activation was determined by measuring NF‐κB activation by reporter assay, and the amount of Interleukin‐8 (IL‐8) secreted by enzyme‐linked immunosorbent assay (ELISA). The data shown are the mean results obtained from 3 independent experiments. ***p < 0.001.

To investigate the effect of *L. reuteri‐F. nucleatum* coaggregates on immune response, PDLF, oral epithelium cells, and human monocytes were infected with *F. nucleatum* alone, unconjugated mixtures of *L. reuteri* and *F. nucleatum* , or *L. reuteri‐F. nucleatum* coaggregates. When cells were infected with unconjugated mixtures of *L. reuteri* and *F. nucleatum*, a significantly increased amount of IL‐8 was produced by PDLF and oral epithelial cells compared to cells infected with *F. nucleatum* alone (Figure 3A,B). A similar trend was observed with monocytes, where infection with *F. nucleatum* alone induced a significant increase in NF‐κB activation and production of IL‐8 (Figure 3C). When *L. reuteri* DSM was coaggregated with *F. nucleatum* before cell infection, a reduced inflammatory response was observed in all 3 cell types compared to *F. nucleatum* self‐aggregates. This reduced inflammatory response was reversed when the coaggregation was disrupted using D‐arginine or galactose, suggesting that bacterial aggregates modulated the host inflammatory responses (Figure 3D–F). Since D‐arginine or galactose alone and in combination with bacteria did not modulate inflammatory response, and the cell infection did not adversely affect viability (Figure S3A–C), we hypothesized that bacterial aggregates reduced immune response by sequestering pathogen‐associated molecular patterns (PAMPs), leading to decreased activation of pattern recognition receptors (PRRs). Since TLR2 and TLR4 are the key cell surface PRRs activated by PAMPs from *L. reuteri* and *F. nucleatum* [17], we tested this hypothesis using the HEK TLR2 and HEK TLR4 cell reporter models. We found that *L. reuteri‐F. nucleatum* aggregates activated NF‐κB less than *F. nucleatum* self‐aggregates in HEK TLR4 cells, and not in the HEK TLR2 cells (Figure 4). Similar results were observed for both DSM and PTA strains. Blocking aggregate formation with D‐arginine or galactose restored immune activation levels to those seen with *F. nucleatum* alone (Figure 4). These treatments did not affect immune response or cell viability (Figure S3D,E). Collectively, these results indicate that coaggregation with *F. nucleatum* , *L. reuteri* DSM, and PTA strains reduced the innate immune response by reducing the activation of TLR4.

### 3.4 Effects of *L. reuteri* on *P. gingivalis* Colonization

**FIGURE 5:**
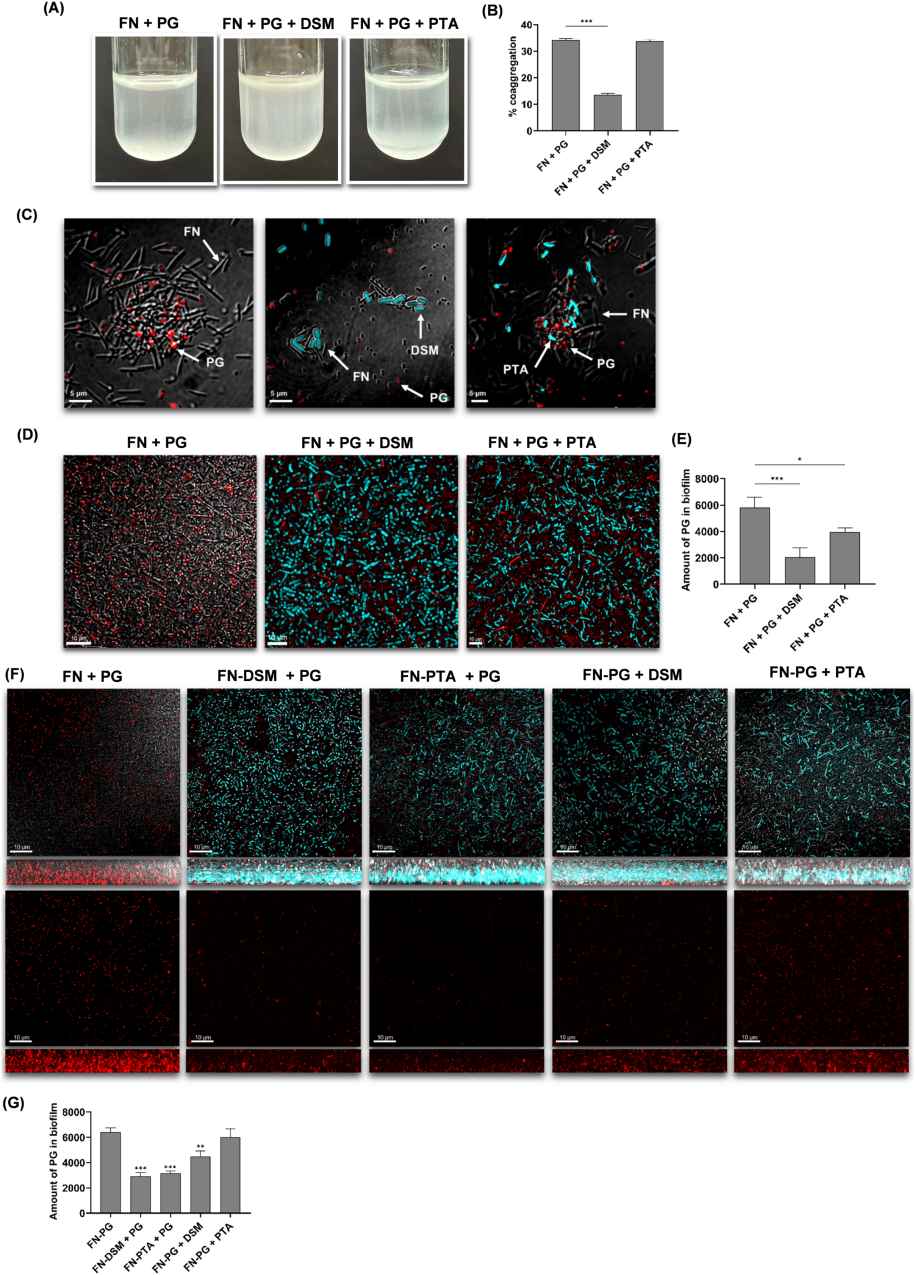
Effects of *L. reuteri* on *F. nucleatum* ‐ *P. gingivalis* interaction. (A) Tube coaggregation assay of *F. nucleatum* (FN) and *P. gingivalis* (PG), FN and PG with *L. reuteri* DSM, and FN and PG with *L. reuteri* PTA. (B) Quantitative analysis of the amount of coaggregates formed. CLSM analyses of (C) bacterial aggregates and (D) dual or tri‐species biofilms. PG was labeled red while DSM or PTA was labeled blue. (E) Quantification of the amount of PG within these biofilms. (F) FN and *L. reuteri* DSM or PTA coaggregates were formed first, and PG was added subsequently (FN‐DSM + PG, FN‐PTA + PG). Alternatively, FN and PG coaggregates were formed first, and *L. reuteri* DSM or PTA was added thereafter. PG was labeled red while DSM or PTA was labeled blue. CLSM images of top and side views are shown. (G) The proportion of PG within these biofilms was quantified using the Imaris 10.1 software. The data shown are the mean results obtained from 3 independent experiments. *p < 0.05; ***p < 0.001.

Since the colonization of *P. gingivalis*, the keystone pathogen, is reliant on *F. nucleatum* [18, 19], we determined whether *L. reuteri* could compete with *P. gingivalis* for the surface adhesins on *F. nucleatum* . In a tube coaggregation assay, we observed that *P. gingivalis* and *F. nucleatum* formed large visible clumps that settled quickly (Figure 5A). With the introduction of *L. reuteri* DSM strain, smaller clumps were formed, whereas *L. reuteri* PTA strain did not affect clump size. When the extent of coaggregation was quantified, a significant reduction in coaggregation formation was observed only with the addition of *L. reuteri* DSM (Figure 5B). These macroscopic observations were corroborated by microscopic findings using CLSM analysis. The addition of *L. reuteri* DSM resulted in its preferential coaggregation with *F. nucleatum* , disrupting the formation of *P. gingivalis–F. nucleatum* coaggregates (Figure 5C). In contrast, *L. reuteri* PTA intermingled with *F. nucleatum* and *P. gingivalis* aggregates.

To assess the integration of *L. reuteri* into *F. nucleatum‐P. gingivalis* biofilms, *F. nucleatum* was co‐cultured with *P. gingivalis* in the presence and absence of *L. reuteri* . Both strains successfully integrated into the biofilm, resulting in a significant reduction of *P. gingivalis* levels in the presence of *L. reuteri* (Figure 5D,E). We explored how the timing of *L. reuteri's* introduction affected *P. gingivalis* incorporation in biofilm. The reduction in *P. gingivalis* was greatest when *L. reuteri* DSM or PTA was present before the formation of *F. nucleatum* and *P. gingivalis* aggregates, with a 50% reduction observed (Figure 5F,G).

## 4 Discussion

**FIGURE 6:**
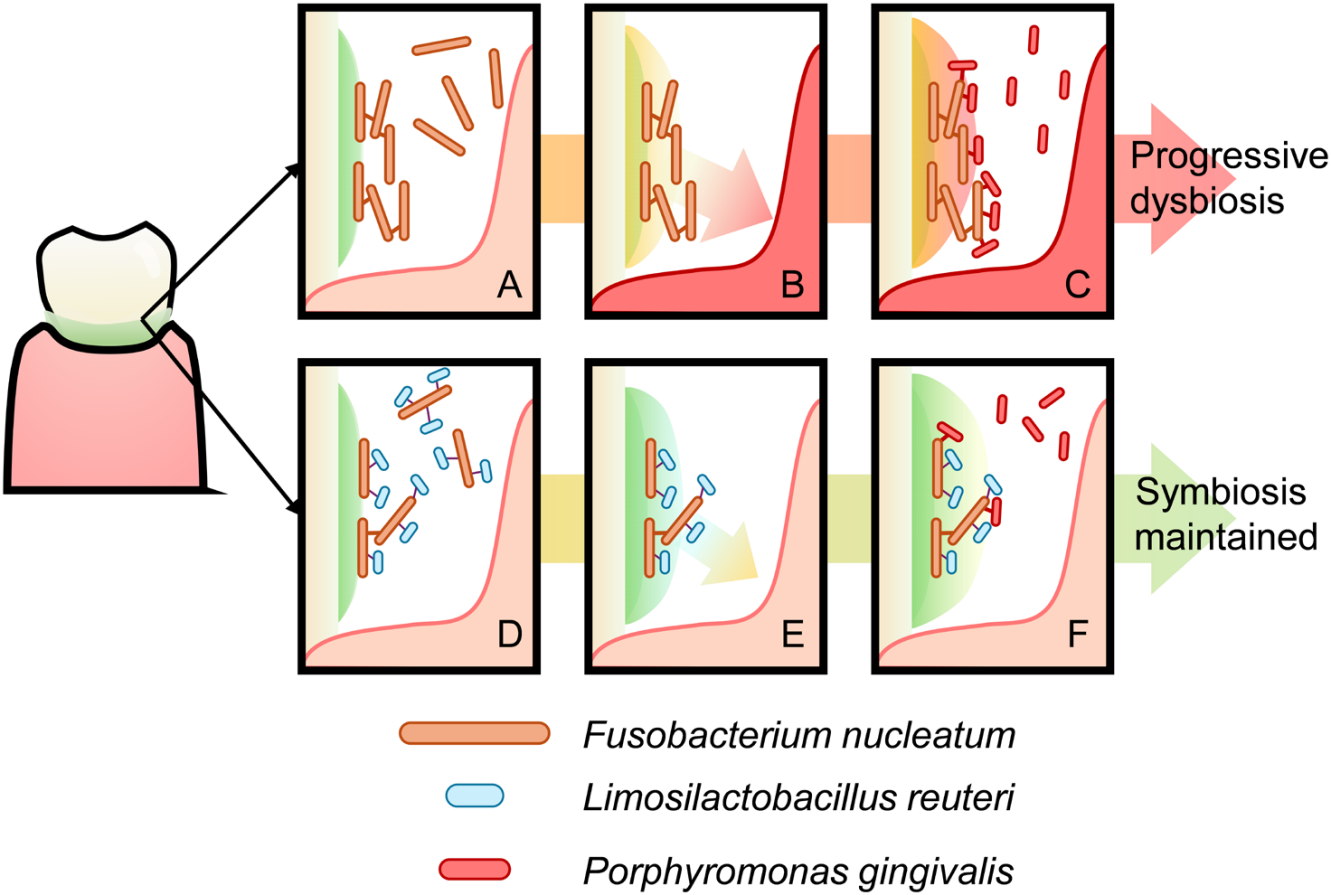
Graphical summary of the findings of this study. *F. nucleatum* plays a key role in the development of the dental plaque biofilm. Without intervention, it colonizes the developing biofilm (A), leading to an overall increase in the immunogenicity of the developing biofilm. (B) The elicited host inflammatory responses then alter the microenvironment within the periodontal pocket, favoring the inflammophilic bacteria. (C) *F. nucleatum* also enables the colonization by *P. gingivalis* , the keystone periodontal pathogen. These changes lead to progressive subgingival microbiome dysbiosis, contributing to progressive periodontitis. Through the coaggregation between *L. reuteri* , the abundance of *F. nucleatum* within the biofilm can be reduced (D), minimizing the immunogenic stimulus on the periodontium. (E) The colonization of *P. gingivalis* can also be mitigated (F), maintaining a symbiotic host‐microbial relation compatible with periodontal health.

In the clinical context of probiotics as adjuncts to nonsurgical periodontal treatment, our limited understanding of their underlying mechanisms may have contributed to suboptimal application, translating to the varying effectiveness observed [4]. Since *F. nucleatum* is the key orchestrator of biofilm development and maturation, this study characterized the interaction between *L. reuteri* and *F. nucleatum* , revealing its implications for biofilm composition and immunogenicity (Figure 6).

*F. nucleatum* is essential for the development of a dysbiotic biofilm. As a bridging species, it facilitates coaggregation with oral streptococci and incorporation of periodontal pathogens like *P. gingivalis* , *Tannerella forsythia* , and *Treponema denticola* [20]. Its large surface area is covered with various adhesins, allowing *F. nucleatum* to interact effectively with numerous bacteria. Without *F. nucleatum* , the abundance of periodontal pathogens in the biofilm is significantly reduced [18], emphasizing its critical role in developing subgingival microbiome dysbiosis. In this study, we found that coaggregation between *L. reuteri* and *F. nucleatum* disrupts the latter's bridging role, reducing its presence in the biofilm and preventing *P. gingivalis* colonization. These changes would influence biofilm development, altering its composition and thus limiting the development of dysbiosis. Our findings also explain the reduced recolonization by *P. gingivalis* in patients treated with adjunctive *L. reuteri* probiotics [6].

However, this mechanism is most effective when *L. reuteri* is administered early, before *F. nucleatum* coaggregates with *P. gingivalis* . If *L. reuteri* is administered after this coaggregation has occurred, its efficacy decreases significantly, with a ~50% reduction observed for *L. reuteri* DSM and *L. reuteri* PTA, losing its ability to prevent *P. gingivalis* colonization. Clinically, these findings underscore the importance of disrupting the existing subgingival biofilm before administering probiotics. The basal abundance of *P. gingivalis* and the extent of its suppression by periodontal treatment likely influence the efficacy of *L. reuteri* , which may explain the variability in clinical outcomes [5]. Additionally, the results also suggest that immediate post‐debridement administration of *L. reuteri* could enhance its performance. Future clinical studies will be required to validate these inferences.

In addition, this study also demonstrated how coaggregation with *L. reuteri* modulated the immunogenicity of *F. nucleatum* . Immunogenicity represents an important phenotypical trait that elicits and perpetuates the host inflammatory response responsible for periodontal tissue destruction, which in turn alters the microenvironment of the periodontal pocket to favor inflammophilic pathobionts [21]. Thus, the ability to modulate microbial immunogenicity will interrupt the reciprocal host–microbe interactions that perpetuate periodontal tissue destruction. While unaggregated forms of *L. reuteri* elicited minimal IL‐8 production by periodontal fibroblasts and oral epithelial cells, they provoked significant immunogenic responses in the monocytes, predominantly via TLR‐2 activation. This basal immunogenicity exhibited an additive effect, resulting in the increased immunogenicity observed for the unaggregated *L. reuteri* ‐ *F. nucleatum* mixtures. In contrast, coaggregation of *L. reuteri* ‐ *F. nucleatum* resulted in significant reductions in immunogenicity, primarily mediated by decreasing TLR‐4 activation. Amongst the PAMPs present in *F. nucleatum* , lipopolysaccharide (LPS), an outer membrane component of gram‐negative bacteria, is a strong instigator of TLR‐4 mediated immune responses contributing to the inflammatory changes of periodontitis [22]. Through coaggregation and the formation of corncob structures, *L. reuteri* may sequester the immunogenic LPS of *F. nucleatum* , preventing its interaction with TLR‐4, thus modulating its immunogenicity and mitigating host inflammatory responses. From a therapeutic point of view, this ability to modulate immunogenicity may be an important mechanism by which probiotics enhance periodontal healing, especially since the overall immunogenicity of the biofilm and the longitudinal profiles of TLR‐4 activation are associated with the outcomes of nonsurgical periodontal treatment [23, 24]. The reduced immunogenicity and thus host inflammatory responses may also explain the benefits observed for bleeding on probing and the gingivitis indices [5]. Considering these benefits of modulating immunogenicity and thus host inflammatory responses, long‐term probiotic use may minimize progressive periodontitis during supportive periodontal treatment.

Prior mechanistic studies on *L. reuteri* have primarily focused on its secreted metabolites, such as acids, reuterin, and hydrogen peroxide [9, 25]. In contrast, our findings suggest a different perspective, where the effects of *L. reuteri* on *F. nucleatum* are mediated through direct cell–cell interactions mediated by surface adhesin‐receptor interactions. While reuterin production by *L. reuteri* was not undetectable using a colorimetric assay, we cannot exclude the possibility that coaggregation facilitates close interactions between *L. reuteri* and *F. nucleatum* such that even minute amounts of reuterin can exert their antimicrobial effects on the latter. In addition, it will be interesting to perform metabolomic or proteomic characterization of the secretome to determine the potential impact of coaggregation on the bacterial secretome. By screening a panel of small molecules, we identified the mechanistic nature of the adhesin‐receptor interaction between *L. reuteri* DSM and *F. nucleatum* , as well as *L. reuteri* PTA and *F. nucleatum* , to be sensitive to D‐arginine and galactose, respectively. These small molecules likely act as competitive inhibitors, binding to the complementary surfaces on the adhesins required for *L. reuteri* – *F. nucleatum* coaggregation, thereby disrupting the process. Upon their addition, the inhibitory effects on *F. nucleatum* were mostly reversed, confirming the mechanistic role of coaggregation in this interaction. These findings are insightful as they imply that these *L. reuteri* strains elicited their effects through binding to different adhesins on *F. nucleatum* .

The ability of *L. reuteri* ATCC PTA 5289 to clump with *F. nucleatum* aggregates may be influenced by its ecological origin. This strain was isolated from the oral cavity of a Japanese woman with good oral hygiene. The oral origin of *L. reuteri* ATCC PTA 5289 may have favored the evolution of compatible adhesin‐receptor interactions that facilitate its integration into *F. nucleatum* aggregates, which likely represent its native biofilm state in the oral environment. On the other hand, *L. reuteri* ATCC DSM 17938 was isolated from the breast milk of a Peruvian mother. These differences in co‐aggregation characteristics with *F. nucleatum* could have contributed to the similarities and differences in the biological effects of the two *reuteri* strains observed throughout the study.

While this study unveiled novel insights into the underlying mechanisms of *L. reuteri* , the limitations of this study should be considered when interpreting these findings. The actual recolonization within the oral cavity is highly complex, involving coaggregation amongst multispecies microbial communities, which was not reproduced here as a more defined approach was needed to address the specific research questions posed in this study. However, given the key role of *F. nucleatum* as a bridging species for periodontal pathogens, its potential impact may be inferred from our findings with *P. gingivalis* , where the efficacy of *L. reuteri* was partly inhibited. These negative effects may be minimized with adequate suppression of the oral microflora and timely administration of *L. reuteri* . The probiotic effects will also require repeated topical exposures through continued consumption of the lozenges, since the presence of *L. reuteri* gradually disappears after completing the course of probiotics [26]. Additional clinical studies will be needed to validate our inferences.

## 5 Conclusion

This in vitro study demonstrated that coaggregation between *L. reuteri* and *F. nucleatum* affects its function as the bridging species, thus altering composition and reducing the immunogenicity of the resulting biofilm. These insights highlight the importance of bacterial coaggregation in mediating the effects of *L. reuteri* as a periodontal probiotic.

## Author Contributions

Ren Jie Jacob Chew and Kai Soo Tan conceived the study. Luming Yang performed the experiments and acquired the data. All authors have been involved in experimental design, data interpretation, drafting of the manuscript, and revising it critically, and have given final approval of the version to be published.

## Conflicts of Interest

The authors declare no conflicts of interest.

## Supporting information


Data S1.

## Data Availability

The data that support the findings of this study are available from the corresponding author upon reasonable request.
