# Supplementary material for: Limosilactobacillus reuteri‐ Fusobacterium nucleatum Interactions Modulate Biofilm Composition and Immunogenicity
Source: J Periodontal Res. 2025 Jul 25;60(10):1006–17. doi: 10.1111/jre.70021 (PMC12640217; doi:10.1111/jre.70021)
Supplement: Supplementary file 1 — Data S1. [file JRE-60-1006-s001.docx]

**Supplementary Materials**

**Materials and Methods**

Bacterial culture

This study used 2 strains of *L. reuteri*, *L. reuteri* DSM 17938 and *L. reuteri* ATCC PTA 5289 (BioGaia, Stockholm, Sweden)*,* and 2 periodontal pathobionts, *Fusobacterium nucleatum* ATCC 25586*,* and *Porphyromonas gingivalis* W50 *(American Type Culture Collection, Virginia, USA)*. *L. reuteri* was cultured in MRS broth (Bio Basic, Singapore). *F. nucleatum* and *P. gingivalis* were cultured in brain heart infusion (BHI) broth (Neogen, Michigan, USA), supplemented with 0.5% yeast extract (Neogen), 0.05% hemin (Merck, Missouri, USA) and 0.0001% menadione (Merck), and incubated in an anaerobic chamber supplemented with 80% N_2_, 10% H_2_, and 10% CO_2_.

Spot assay

*S. oralis* ATCC 6249 and *S. mitis* ATCC 49456 were maintained on BHI agar plates (Neogen). Overnight bacterial cultures were prepared by inoculating an isolated colony in BHI broth (Neogen), and incubated aerobically at 37°C with 5% CO_2_. Positive control groups consisted of 7μL of 1 x 10^9^ CFU/mL of either *S. mitis* or *S. oralis* spotted on BHI agar plates and incubated under aerobic or anaerobic conditions for 24h. Thereafter, *F. nucleatum* was spotted and incubated anaerobically for 24h. Control groups which consistent of *F. nucleatum* spotted on agar and incubated aerobically and anaerobically for 24h was also included.

Reuterin assay

The production of reuterin was assessed by a quantitative colorimetric assay modified from(Cadieux et al., 2008; Navarro, Mashburn-Warren, Bakaletz, Bailey, & Goodman, 2017). *F. nucleatum* biofilms were inoculated at 1 × 10^7^ CFU and cultured together with *L. reut*eri DSM or PTA strains at a ratio of 1:1 and 1:5 in 24-well cell culture plates (Thermo Fisher Scientific). Biofilms were incubated anaerobically at 37°C. Positive control groups included *L. reuteri* supplemented with 2% v/v glycerol. After 24h incubation, supernatant of biofilm was collected and passed through a 0.2μm pore size syringe filter. Quantification of reuterin was performed as described previously. A 100μL supernatant/standard was added to 75μL of tryptophan solution (0.01M tryptophan in 0.05M HCl). Then, 300μl of ice-cold concentrated HCl was added, and the solution was incubated in the dark for 20min at 37°C. Absorbance at 560nm was measured using a microplate reader (Biotek). The amount of reuterin present in the culture supernatant was determined using a standard curve generated using reuterin (MCE, MedChemExpress).

Crystal violet assay

Crystal violet (CV) assay was carried out to quantify the amount of biofilm biomass. First, the culture supernatant was removed gently and the biofilm was gently washed once with sterile 1× PBS. Thereafter, biofilms were fixed with methanol (Merck) for 10 min after which methanol was removed and the wells air dried. The biofilms were stained with 0.1% CV (Merck) solution for 15min with shaking at 100rpm. Subsequently, the wells were rinsed with distilled water to remove excess dyes. Finally, the dye was dissolved with 1mL 33% v/v acetic acid (Merck). The absorbance was measured at 580nm using a microplate reader.

Coaggregation assay

Quantitative coaggregation assays between live and heat-killed *L. reuteri* DSM or PTA strains and *F. nucleatum* were investigated. In addition, coaggregation assays between live *L. reuteri* and *F. nucleatum* were determined in the presence or absence of the small molecule of interest listed in Supplementary Table 1. The tested small molecule was dissolved into coaggregation buffer and adjusted pH to 7.4 before mixing the tested paired bacterial species into a clear glass test tube for coaggregation.

**Supplementary Table 1.** List of molecules tested for the effects on coaggregation between *L. reuteri* and *F. nucleatum*.

| Small molecule | Concentration | Source | Reference |
| --- | --- | --- | --- |
| Glucose | 50mM | Merck | (Kinder & Holt, 1989) |
| Sucrose | 50mM | Merck | (McIntire, Vatter, Baros, & Arnold, 1978) |
| Galactose | 50mM | Merck | (Coppenhagen-Glazer et al., 2015) |
| D-arginine | 50mM | Merck | (Alvarez, Aliashkevich, de Pedro, & Cava, 2017) |
| L-arginine | 50mM | Merck | (Kaplan, Lux, Haake, & Shi, 2009) |
| EDTA | 50mM | Merck | (Taweechaisupapong & Doyle, 2000) |

Cell culture conditions

Primary human periodontal ligament fibroblasts, PDLF, (Science, California, USA), a human oral epithelial cell line, TR146, (European Collection of Authenticated Cell Cultures, Salisbury, United Kingdom), were cultured in Dulbecco's Modified Eagle Medium, DMEM (Cytiva, Utah, USA) supplemented with 10% fetal bovine serum (FBS).

The human monocytic cell line THP-1 BLUE^TM^ stably expressing the NFκB-SEAP reporter (Invivogen, California, USA) was cultured in RPMI 1640 (Cytiva) supplemented with 10% FBS. The HEK TLR2 and HEK TLR4 stably expressing the NFκB-SEAP reporter (Invivogen) were cultured using DMEM (Hyclone) supplemented with 10% FBS. All cell types were incubated at 37°C in a humidified incubator supplemented with 5% CO_2_.

Cell viability assay

The viability of cells were determined by the trypan blue exclusion assay. Equal volumes of 0.4% trypan blue (Merck) was added to the cell suspension, and the number of viable cells were enumerated using a hemocytometer. The proportion of viable cells was expressed as a percentage of the total number of enumerated cells.

**Results**

**
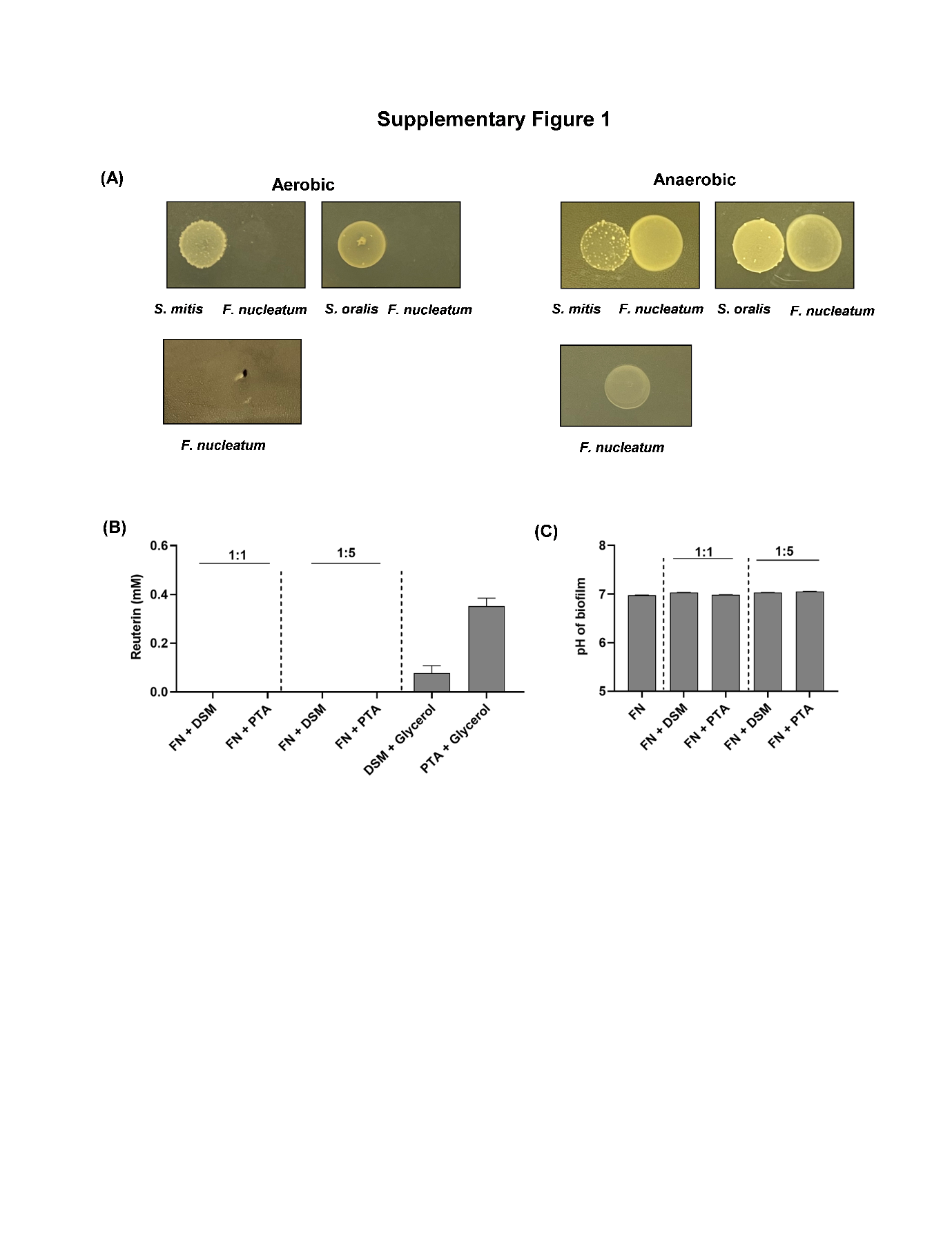
**

**Supplementary Figure 1.** (A) *S. mitis* or *S. oralis* was spotted on BHI agar plates and incubated aerobically or anaerobically for 24h. Thereafter, *F. nucleatum* (FN) was spotted and incubated anaerobically for 24h. *S. mitis* and *S. oralis* inhibited the growth of *F. nucleatum* under aerobic but not anaerobic conditions. This was likely attributed to hydrogen peroxide produced during the oxidative metabolism of *S. mitis* and *S. oralis* (Tang, Sim, & Tan, 2022). FN spotted on agar and incubated aerobically or anaerobically for 24h was also included as a control. (B) FN was co-cultured with *L. reuteri* DSM or PTA strains at a ratio of 1:1 or 1:5 (*FN:L. reuteri*) anaerobically for 24h. Thereafter, the amount of reuterin in the culture supernatant was determined via colorimetric assay. (C) pH of biofilms was determined using a pH microprobe.


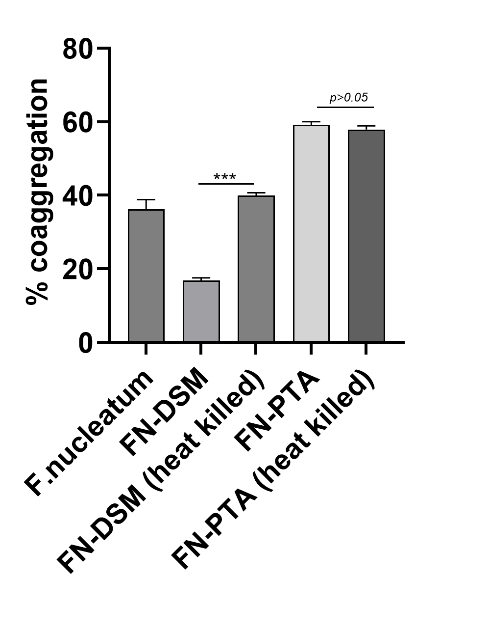


**Supplementary Figure 2.** Quantitative co-aggregation experiments were carried out between *F. nucleatum* (FN) alone, FN and live *L. reuteri* or heat-killed *L. reuteri* strains. ****p<0.001*

**
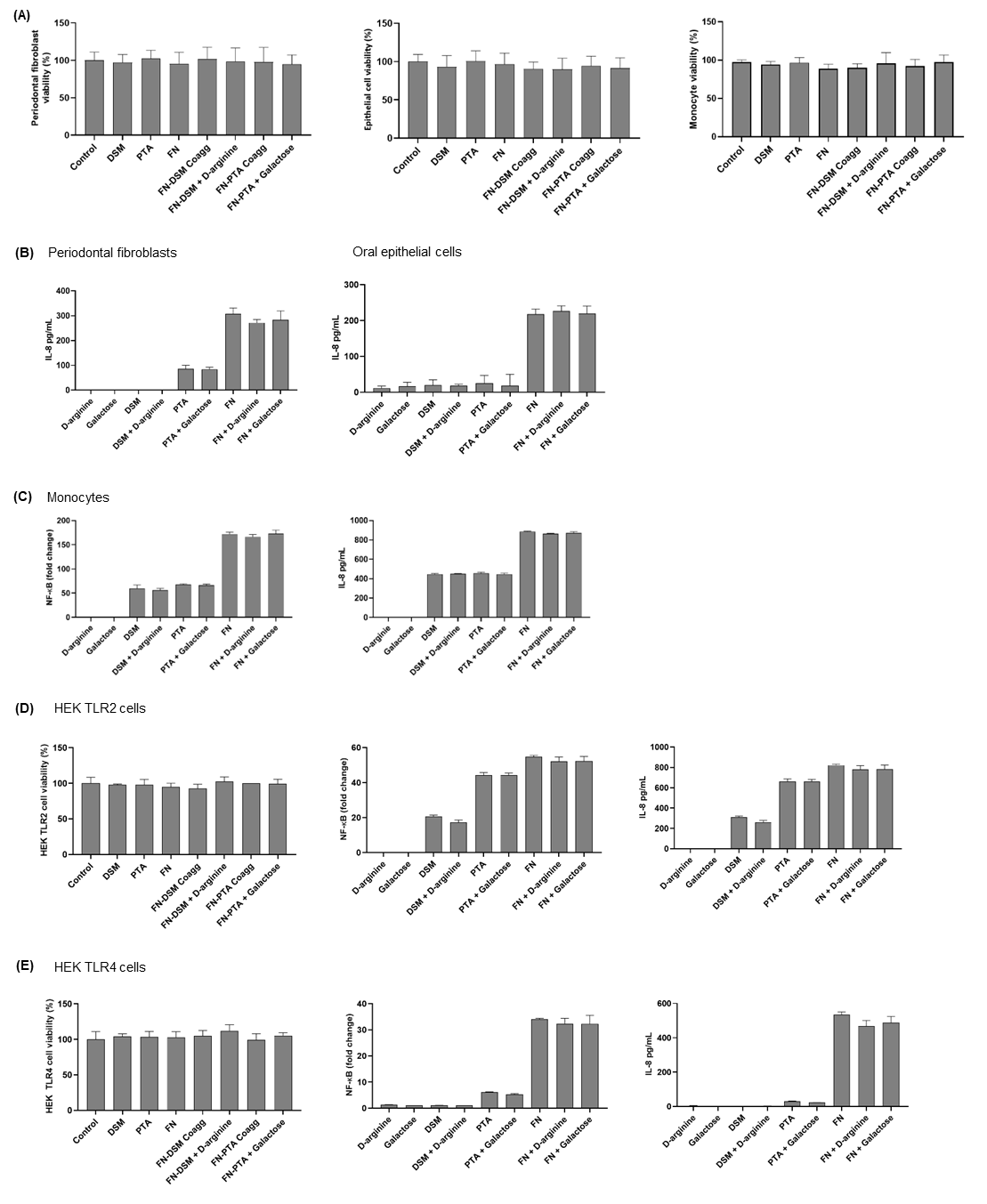
**

**Supplementary Figure 3.** The viability of (A) periodontal fibroblasts, oral epithelial cells, and monocytes were determined by trypan blue dye exclusion assay. Amount of IL-8 secreted by (B) periodontal fibroblasts and oral epithelial cells following treatment with the indicated agents for 6h. (C) NF-κB activation and IL-8 production by monocytes following treatment with the indicated agents for 6h. Viability and amount of NF-κB activation and IL-8 secretion by (D) HEK TLR2 or (E) HEK TLR4 cells.

**References**

Alvarez, L., Aliashkevich, A., de Pedro, M. A., & Cava, F. (2017). Bacterial secretion of D-arginine controls environmental microbial biodiversity. *The ISME Journal, 12*(2), 438-450. doi:10.1038/ismej.2017.176

Cadieux, P., Wind, A., Sommer, P., Schaefer, L., Crowley, K., Britton, R. A., & Reid, G. (2008). Evaluation of reuterin production in urogenital probiotic Lactobacillus reuteri RC-14. *Appl Environ Microbiol, 74*(15), 4645-4649. doi:10.1128/AEM.00139-08

Coppenhagen-Glazer, S., Sol, A., Abed, J., Naor, R., Zhang, X., Han, Y. W., & Bachrach, G. (2015). Fap2 of Fusobacterium nucleatum is a galactose-inhibitable adhesin involved in coaggregation, cell adhesion, and preterm birth. *Infect Immun, 83*(3), 1104-1113. doi:10.1128/iai.02838-14

Kaplan, C. W., Lux, R., Haake, S. K., & Shi, W. (2009). The Fusobacterium nucleatum outer membrane protein RadD is an arginine-inhibitable adhesin required for inter-species adherence and the structured architecture of multispecies biofilm. *Mol Microbiol, 71*(1), 35-47. doi:10.1111/j.1365-2958.2008.06503.x

Kinder, S. A., & Holt, S. C. (1989). Characterization of coaggregation between Bacteroides gingivalis T22 and Fusobacterium nucleatum T18. *Infect Immun, 57*(11), 3425-3433. doi:10.1128/iai.57.11.3425-3433.1989

McIntire, F. C., Vatter, A. E., Baros, J., & Arnold, J. (1978). Mechanism of coaggregation between Actinomyces viscosus T14V and Streptococcus sanguis 34. *Infect Immun, 21*(3), 978-988. doi:10.1128/iai.21.3.978-988.1978

Navarro, J. B., Mashburn-Warren, L., Bakaletz, L. O., Bailey, M. T., & Goodman, S. D. (2017). Enhanced Probiotic Potential of Lactobacillus reuteri When Delivered as a Biofilm on Dextranomer Microspheres That Contain Beneficial Cargo. *Front Microbiol, 8*, 489. doi:10.3389/fmicb.2017.00489

Tang, Y. L., Sim, T. S., & Tan, K. S. (2022). Oral streptococci subvert the host innate immune response through hydrogen peroxide. *Sci Rep, 12*(1), 656. doi:10.1038/s41598-021-04562-4

Taweechaisupapong, S., & Doyle, R. J. (2000). Sensitivity of bacterial coaggregation to chelating agents. *FEMS Immunology & Medical Microbiology, 28*(4), 343-346. doi:10.1111/j.1574-695X.2000.tb01496.x
